# Create Machine Vision Inspired by Eagle Eye

**DOI:** 10.34133/2022/9891728

**Published:** 2022-06-01

**Authors:** Haibin Duan, Xiaobin Xu

**Affiliations:** ^1^ State Key Laboratory of Virtual Reality Technology and Systems, School of Automation Science and Electrical Engineering, Beihang University (BUAA), Beijing 100083, China; ^2^ Peng Cheng Laboratory, Shenzhen 518055, China

## Abstract

Eagle, a representative species in the raptor world, has the sharpest visual acuity among all animals. The reputation of the “clairvoyance” is employed to describe an eagle. The excellent visual skills of eagles depend on their unique eye structures and special visual principles. The powerful vision perception mechanisms of the eagle bring abundant inspiration for traditional visual applications. Biological eagle eye vision technology provides a creative way to solve visual perception issues of “Knowing What is Where by Seeing.” The theoretical research and practical works of eagle vision would contribute to the development of machine vision, or even artificial intelligence (AI) in the real world. Furthermore, eagle eye vision also provides feasible ideas for the popularization of new concepts in the virtual world in the future.

## 1. Introduction

About 540 million years ago, creatures evolved the eye [ 1– 3]. More than 80% information of the objective world is perceived through the eyes. Since then, the activity mode and survival law of creatures have changed [ 4]. Eagles have evolved the sharpest vision among all animals in the long process of evolution [ 5]. The fast and accurate visual information processing abilities of eagles are lost in wonder, which mainly benefits from unique physiological structures and visual processing mechanisms. Using optical devices to imitate the physiological structures of eagle vision is important to improve the performance of hardware devices. Besides, some visual processing procedures of eagles are closely related to computer vision processing technology, especially in high dynamic, high precision, large view field, and high complexity mission environment. More importantly, machine vision is an essential branch of the artificial intelligence (AI) field in the real world. Studying and simulating both the special structures and mechanisms of the eagle eye open up a new train for the development of machine vision. Furthermore, mechanisms of eagle vision can be imported to the virtual world to enhance the security, availability, and experience of the virtual space.

## 2. Unique Physiological Structure

Eagle vision system is so outstanding that the prey can be locked and tracked outside several kilometers. The phenomenon is inseparable from the unique physiological structure of the eagle eye. Heavily visual-dependent species and highly sensitive organisms have larger eye sizes [ 6]. Eagle eyes account for 15% of the weight of their head, while that of humans accounts for only 2% [ 7]. Therefore, the area of an image projected on the retina is increased because of the big tubular eyes of eagle [ 8, 9]. The photoreceptor cells on the eagle retina can reach up to 1 million/mm ^2^ (that of human is 200 thousand/mm ^2^), so the spatial resolution is higher [ 10]. Eagle eye’s high spatial resolution can be employed in scenes that pay more attention to detail detection, including intelligent medical treatment, defect detection, and aerial image analysis. Each eye of eagle includes two foveas: median fovea (deep fovea) and lateral fovea (shallow fovea) [ 9]. Median fovea, used for monocular vision, is more suitable for objects at long distances. The lateral fovea plays a major role in binocular vision for observing objects at close distances. Two foveas cooperate to adapt to the hunting scene [ 11]. Monocular vision and binocular vision constitute the total view field of eagle [ 12]. Raptors have different view fields due to the influence of their living environment [ 13]. The view field of eagle covers 260° and 80° in horizontal direction and vertical direction, respectively [ 14]. Notably, the large view field structure of eagle provides a new solution for designing a panoramic camera. Based on the above features of eagle eye, Deng and Duan invented an integrated variable resolution imaging device [ 15].

Eagles have excellent color recognition ability and light adaptability. The main structures related to the above abilities include photoreceptors, pecten, and oil droplets. The photoreceptors on the retina of eagle include cone cells and rod cells [ 16]. Cone cells, only located in the fovea, are sensitive to strong light and colorful colors. Rod cells, distributed around the fovea, are related to weak light and achromatic color [ 17]. Oil droplets (red, green, blue, yellow, and colorless) with a high concentration of carotene are distributed at the end of cone cells [ 18]. The proportion of oil droplets adjusts according to the living environment. Besides, Seifert et al. suggested that the distribution of cone cells is hyperuniformity, even more perfect than it [ 19]. It may be the reason why birds have excellent vision, especially the eagle.

## 3. The Impeccable Optic Nerve System

The process of the vision information processing is inseparable from the participation of the optic nerve system of eagle [ 20– 22]. The visual nervous system of eagle mainly contains four pathways: thalamofugal pathway, tectofugal pathway, retinofugal pathway, and accessory optic system [ 23]. Four pathways are complementary and indispensable. After decades of research on anatomy, biophysics, and neuroethology, it has shown that these capabilities of color discrimination, luminance adaptation, shape recognition, and moving target detection of eagle are invalid without the visual nervous system [ 24].

Optic tectum, an important part of the tectofugal pathway or midbrain, is the visual center of eagle for fusing and transmitting vision information. Optic tectum and nucleus isthmi together comprise competition and selection branch for input visual stimulations in the tectofugal pathway [ 25]. Visual attention mechanism, lateral inhibition mechanism, and winner-take-all mechanism occurred in optic tectum-nucleus isthmi pathway [ 26] are investigated. These mechanisms of eagle vision have been applied in target detection, target tracking, contour extraction, autonomous air refueling, and autonomous carrier landing.

Moving object detection is also closely related to the optic nerve system of eagle. Moving target detection mainly focuses on two aspects: fault-tolerant ability and optical flow sensitivity. Parallax sensitive cells, accommodating a certain range of visual noise, exist in the visual cortex of eagle. Eagle is expert in observing and estimating the movement of prey through optical flow. Therefore, eagle can accurately find, track, and capture prey in complex and variable scenes.

## 4. Future Eagle Vision Applications

**Figure 1:**
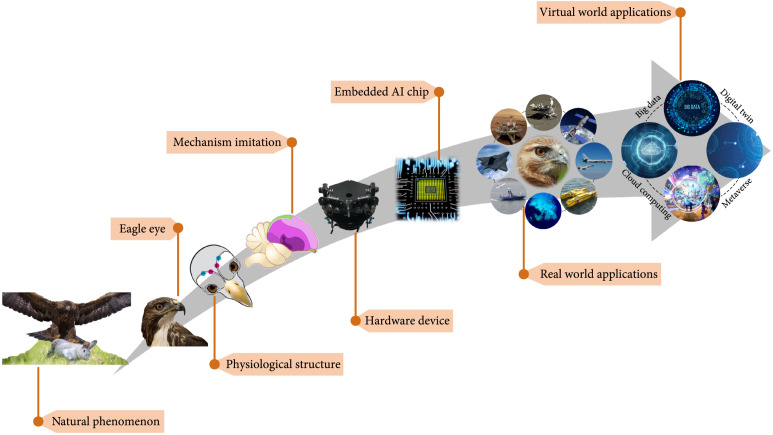
Development route of the biological eagle vision. Starting from the predator-prey phenomenon of eagle in nature, the physiological structures and the eagle vision mechanisms will be investigated and imitated. A preliminary hardware device is invented to simulate the characteristics of eagle eyes. Embedded AI chips should be further designed. The complete eagle vision system is expected to be applied in real world and virtual world.

Vision technology is a critical component of intelligent machine or AI. Machines with keen vision system can commendably accomplish complex, dangerous, and tedious tasks in various environments. In view of the above requirements, machine vision puts forward higher standards for accuracy and adaptability. The exploration of eagle vision mechanisms can provide an effective development route for modern machine vision (Figure 1). The typical contents of the route mainly include physiological structure, biological mechanism, computational models, hardware device and microchips, and practical applications. Moreover, eagle vision can also bring extraordinary experience for human society with the digital age coming.

### 4.1. Investigation of the Biological Mechanism

Some mechanisms based on eagle vision were established in the past years, such as contrast sensitivity mechanism, color antagonism mechanism, visual attention mechanism, and lateral inhibition mechanism [ 27]. The above mechanisms of eagle vision provide feasible solutions for machine vision. As the foundation of eagle vision technology, the physiological structure and functional characteristics of eagle eye are expected to be further investigated. Furthermore, the function and mechanism of different information processing units for biological eagle eye vision system should be analyzed to improve the detection accuracy and speed of machine vision in complex and dynamic mission circumstances.

The exploration of undiscovered eagle vision mechanism has a significant effect for developing the biological eagle vision mechanism continuously and deeply. The visual signal processing of eagle eye requires the joint participation of the eye, optic nerve, and brain. For this reason, the only way which must be passed is to establish a comprehensive and integrated eagle eye-nerve-brain visual information processing system model for the research of eagle vision. Therefore, on the premise of complying with the restrictions of animal protection policies, it is necessary to continue the relevant behavioral experiments of eagle eye with the form of minimally invasive by cooperation in various fields.

### 4.2. Computational Physiological Model

The computational physiological model is highly expected to be established using the information processing mechanism of eagle vision [ 28]. The typical models include the retinal structure model, the oil adaptive distribution model, and the receptive field model [ 26]. Using these models to simulate the information processing mechanisms of eagle vision is critically beneficial to applying these mechanisms in improving machine vision. Additionally, as is well-known, the visual pathway of eagle midbrain is an important part in the eagle vision system. Therefore, through analyzing the relationship between the brain pathway and the visual nucleus, the eagle vision-brain mechanism model could be fundamentally built, which can play a significant role in perfecting the eagle eye mechanism model.

### 4.3. Multidisciplinary Study in Eagle Eye-Brain-Cognition-Behavior Technology

The development of biological eagle vision is still in its infancy. The existing information processing mechanisms of eagle vision system are mainly skilled in long-distance small target searching, large view field target tracking, and strong night vision detection. Eagle eye-brain-cognition-behavior mechanism is the complement and improvement for current mechanisms [ 26]. It requires the joint participation of multidisciplinary, including zoology, biology, biophysics, biochemistry, neurology, ethology, optoelectronics, anatomy, physics, electronics, cybernetics, and engineering [ 29]. With the rapid development of AI, machine vision based on biological eagle eye will have characteristics of autonomous learning, data sharing, and human-computer cooperation. Biological eagle vision has important enlightening significance for the diversified development of modern machine vision.

### 4.4. Eagle Vision-Based Devices and Microchips

Biological eagle eye can be simulated from physiological structures or functions. Some sample hardware devices have been invented to simulate the large view field characteristic and variable resolution characteristic of eagle [ 30– 33], and some of the devices are applied in autonomous aerial refueling, robot swarm formation, missile remote guidance, and smart city. However, the combination of eye-brain-cognition-behavior mechanism and microchips is still on the road. Miniaturization is the premise for improving the utilization rate of biological eagle eye devices. Microchips that load with eagle vision mechanisms are crucial to the development of the machine information industry. Besides, the bionic inventions inspired by eagle’s claws and wings are important for farmland protection, camouflage reconnaissance, search, and rescue, which is also a promising way for biological eagle eye device and microchip design.

### 4.5. Cross-Domain Applications Based on Eagle Vision in the Real World

In the real world, highly intelligent machines can be regarded as the most ideal tools for enriching human behaviors. Generally, high-resolution and precise vision is one of the important evaluation indexes of machine intelligence [ 34]. Biological eagle vision is an important technical means to promote the development of machine vision or AI. The technology can be applied for smart transportation, smart agriculture, smart medical, smart catering, geological exploration, and other civil and military industries. Cross-domain [ 35] applications mainly focus on the combination ability and cooperation ability between different spaces and individuals. The performance of the whole system will be significantly improved when each machine in different domain has “smart eagle eye.” Particularly, eagle vision may also have important application value on the land, in air, in ocean and deep sea, and also in space integrative tridimensional military field, all of them are challenging areas for machine vision technologies.

### 4.6. Eagle Vision-Based Applications in the Virtual World

Recently, metaverse [ 36] is a hot point in virtual reality, which is a 1: n projection from the real world to the virtual world. Metaverse emphasizes various experiences and interactions of virtual scenes and describes the virtual world through real logic or imaginary logic [ 37, 38]. Eagle is not only the king of the sky but also the king of vision. If human or machine has a pair of “eagle eyes,” it can capture not only the details of the scenery in front of it, but also the scenery thousands of miles away with wide vision field. Moreover, it will experience the beauty of four-color space. Objects in the scene can be seen even in the weak light environment. Then, it will experience extraordinary scenes without visual blind spots in the virtual world.

The excellent vision of eagle has attracted many biologists for a long time. And many scientists and researchers from other fields also paid abundant attention to this cross-domain field. Biological eagle vision technology provides an effective approach for the enable the modern machine vision or unfolding AI with a more bright and smart “eye.”
